# Towards meeting the research needs of Australian cancer consumers

**DOI:** 10.1186/1756-0500-5-667

**Published:** 2012-12-03

**Authors:** Carla Saunders, Sally Crossing

**Affiliations:** 1 School of Medicine and Public Health, The University of Newcastle, Wallsend, NSW, 2287, Australia; 2 Cancer Voices NSW, PO Box 5016, Greenwich, NSW 2065, Australia

## Abstract

**Background:**

There is a growing amount of literature to support the view that active involvement in research by consumers, especially informed and networked consumers, benefits the quality and direction of research itself, the research process and, most importantly, people affected by cancer. Our exploratory project focuses on identifying their priorities and developing a process to assess the research needs of Australian cancer consumers which may be useful beyond the cancer scenario.

**Methods:**

This project was consumer initiated, developed and implemented, with the assistance of a leading Australian cancer consumer advocacy group, Cancer Voices NSW (CVN). Such direct involvement is unusual and ensures that the priorities identified, and the process itself, are not influenced by other interests, regardless how well-intentioned they may be. The processes established, and data collection via a workshop, followed by a questionnaire to confirm and prioritise findings, and comparison with a similar UK exercise, are detailed in this paper.

**Results:**

Needs across five topic areas reflecting cancer control domains (prevention and risk; screening and diagnosis; treatment; survivorship; and end of life) were identified. Cancer consumers high priority research needs were found to be: earlier diagnosis of metastatic cancers; the extent of use of best practice palliative care guidelines; identifying barriers to cancer risk behaviour change; and environmental, nutrition and lifestyle risk factors for people with cancer. A process for identifying consumers’ research priorities was developed and applied; this may be useful for further investigation in this under-studied area.

**Conclusion:**

The findings provide a model for developing a consumer derived research agenda in Australia which can be used to inform the strategic direction of cancer research. Consumers have been seeking a workable method to achieve this and have worked in collaboration with a major cancer charity, which funds research, to do so.

## Background

Research is central to improving health and contributing to overall national development. Australia has made substantial investments in building and enhancing its health research capacity in recent years. Despite these efforts and some notable examples of success [1,2], the overall picture of progress is a mixed one. Many investments have failed to result in a positive impact on health policies and practices.

No public research funding program can afford to service all potential health research questions, each program must identify priority areas and questions [3]. Within the limits of available funding, it is mostly up to researchers and funding bodies to decide what research to perform within a very broad scope of fundable health research [4]. Yet as Australians experience ever-increasing levels of multiple chronic illnesses and a range of new infectious diseases such as the H1N1 virus [5], decisions on research pursuits require judgments that are more firmly established on the people’s health needs.

Internationally, there is an increasing call for public involvement in identifying health research priorities including distinguishing where and why resources should be allocated [5]. Greater requirements for need-driven versus science-driven research substantiate the value of studies where consumers have helped to identify relevant research questions [6-13] and prioritise topics for the research agenda [8,10,12,14]. Consumer groups such as Cancer Voices NSW (CVN), publish their members' interest in involvement in research and research priorities on their websites [15,16]. Research has found that when potential end recipients assist determinations of research topics, the research results are more likely to respond directly to a prevailing problem and to be utilised in the real world [10,17,18].

There is currently a limited understanding of the research needs of people affected by cancer in Australia. Moreover, published projects that specifically determine needs and gaps are few [19-21]. There is no consensus on the best methods of prioritising health research generally or for cancer research particularly.

A previous UK study [22] found the most highly ranked priority areas were found to be: the impact cancer has on life; how to live with cancer and related support concerns; risk factors and causes of cancer; and early detection and prevention. The investigators describe how the participants believed the emphasis in cancer research had centred on developing treatments and the experience of managing the impact of cancer on individuals was felt to have been relatively neglected. They found that the participants felt that medical research was too focused on discovering new treatments for cancer and they felt a balance in research effort was needed so that the personal consequences of cancer and its treatment were also addressed.

This project set out to investigate the views of those affected by cancer in New South Wales, Australia on what they consider should be the key priorities for future research in this area. The purpose of the paper is to provide information on the research needs of NSW cancer consumers and describe the priority setting process so that interested parties may duplicate the approaches used. The findings, key issues and challenges in the identification and utilisation of consumer research needs are discussed.

## Methodology

This exploratory project adopted a participatory approach, where project ownership was shared with people affected by cancer. Cancer Council NSW (CCNSW), a leading non-government cancer charity and research funder, partnered with Cancer Voices NSW to design and implement an approach to capture cancer consumers’ research needs. CVN, as a peak Australian cancer consumer group with members from all areas of the state of New South Wales had identified this work as a pressing need. Ethical approval was received from the CCNSW Ethics Committee.

Constantly communicating with members is a critical, ongoing aspect of the work of CVN. The organisation routinely surveys its members to ensure an up-to-date understanding of the perceptions and experiences of cancer consumers and to support decisions that will best help people affected by cancer. CVN uses knowledge acquisition to support member empowerment and information, and inform broader policy and advocacy processes.

### Workshop

#### Participants

Cancer consumers, including those impacted by the disease directly, or indirectly as friends or family members, were invited to participate through the following channels: (1) CCNSW website and constituent mail-outs, (2) CVN’ s newsletter and networks, (3) cancer support group contacts and cancer care centres, and (4) via a range of cancer charities and consumer groups. The opportunity was also advertised in the volunteer section of a large, well known recruitment website.

#### Data collection

The methods used to capture consumer research needs were developed after careful deliberation of the support, funding and timeframe within which we were required to work. A workshop designed broadly on the ‘*Global Cafe*’ approach, which had been recommended by CVN, was the main data collection method selected in this part of the project. The Global *Café* process is a technique for harnessing group experience and views on a number of topics, and channelling this output into a usable data set [23].

Data collected from CVN’s own bi-annual survey on cancer research needs was included with an invitation to all participants. They were also asked to provide written input from their constituencies which was incorporated within the pre-workshop data to provide a robust list of consumer research needs.

Prior to and at the commencement of the workshop, participants were provided with information to clarify their general understanding of cancer research across the cancer control spectrum from cancer prevention to screening, diagnosis, management, survival and end of life care. This was designed to focus thinking about cancer research and lead to more informed discussions of what should be researched. Each of these five topic areas became the individual focus of table discussions in the workshop. Participants at each table were asked to discuss the topic as a group, giving their rationale for research interests and then record those research topics they felt to be important on ‘post-it’ notes.

Ideas and conversations of no more than eight participants at each table were linked and built upon as people moved to different tables, cross-pollinating ideas for each topic area and identifying research needs that mattered to their own lived experiences and/or those collected by their consumer network or group. The living network of conversations evolved through several rounds of exploration and research question identification until all participants had the opportunity to input into each of the five topic areas. A cancer researcher or CCNSW research program staff member acted as an advisor on each table for participants’ questions, and if participants required information on research currently being undertaken in the area. The advisors brought discussions back to the issue of research needs if discussions began to go ‘off track’. A pre-selected consumer table host remained at each table and briefed new table arrivals on previous discussions to ensure each new group built on the topic in an iterative exchange process. The needs identified through the small group process were then fed back by the consumer host to the whole group and discussed. This enabled an effective summary of the discussion and also permitted any additional views not covered in the small group to be raised.

### Questionnaire

To verify the workshop results and achieve higher quality results CVN canvassed their membership of over 300 consumers who had been affected by cancer or were interested in cancer control via a general postal questionnaire. A few had attended the workshop. The questionnaire, planned and developed by SC and CS, and tested for readability and ease of understanding by another member of CVN, invited personal reflection on the research needs of consumers. Included with the questionnaire was an explanation of the workshop process and results to ensure an understanding among members of the context within which the questionnaire was sent. CVN members were asked whether their needs reflected some or all of those identified in the workshop. Members were also asked to rate their research needs from 1 to 5, with 1 being their highest research priority. They were asked to identify 5 priorities in each section.

Members were asked to send their responses back to CVN in the included stamped, return addressed envelope. Details of the process steps employed in the project are provided below.

### **Process steps employed in the project**

Steps in the consumer research needs and priorities identification process

1. The requirement for cancer consumer involvement in identifying research needs is established by consumers and acknowledged by professional partners who commit resources and coordination support to the process.

2. A project reference group is established to support the design and implementation of the consumer research needs identification process.

3. Consumer research needs identification approach is clarified and agreed by all parties.

4. Participant recruitment is planned and carried out.

5. Global Café workshop process is developed in detail by the project reference group.

6. The information needed for the workshop is identified and assembled, such as on the cancer control research domains, to help inform participants’ discussions during the workshop and help them build on existing knowledge during their exchanges.

7. Pre-workshop input from members of each consumer group represented at the workshop and the results of previous Cancer Voices NSW survey results are collected for incorporation with the data and information obtained in the workshop.

8. Workshop conducted as planned using the Global Café process.

9. The research needs of cancer consumers are confirmed at the end of the workshop. Follow-up via an emailed report of the workshop outcomes is undertaken to ensure that the final research needs truly reflect the views and advice expressed during the workshop.

10. Workshop findings are strengthened via CVN postal questionnaire to members.

11. The final identified priority issues are compared with a similar project to support the generalisability of the results and help ensure that the priorities are not just intrinsic to this project’s particular process or participants

12. A final report is disseminated to consumer groups, research funding and research organisations

### Data analysis

#### Workshop

Post-it note entries, which were automatically categorised within each of the five cancer control research areas discussed by the workshop table groups, were examined. Repeating issues were identified and summarised. An independent assessment of all data by two members of the project team (CS and SC) was undertaken to ensure consistency in the interpretation of the data.

### Questionnaire

Member priorities were analysed using SPSS version 16 for Windows [24] and are presented as frequencies. Open ended responses were examined to identify which cancer control research domains they belonged to and then individual responses were classified according to existing themes.

## Results

### Workshop

Thirty two people with direct experience of cancer across different cancer types responded to the invitation to participate in the workshop. This number was accepted as workable for the purpose and the process. Participants ranged in age from 18 to 72 years. More females (n=18) than males (n= 14) attended the workshop. Consumers identified needs across the five cancer control research domains with cancer treatment drawing the greatest number of identified issues. Needs across the domains (prevention and risk; screening and diagnosis; treatment; survivorship; and end of life) ranged from a better understanding of unhealthy behaviours such as smoking and sun exposure, to researching factors related to differences in cancer outcome due to personal and geographical inequity, and better understanding the cancer control needs of vulnerable groups such as older people, culturally and linguistically diverse and indigenous people. Improvements in health systems and professional capacity to better meet the needs of the population and quality of life issues for cancer patients, carers and significant others were also deemed to be important areas for inquiry.

An overview of the identified needs within the cancer research domains from which they were raised are provided below.

### **Cancer consumer research needs across the cancer control spectrum**

Prevention and risk

• How can media improve understanding (and behaviour change) of cancer risk factors linked to personal behaviours, with attention to confused use of relative and absolute risk messages?

• Definitive research into nutrition and other lifestyle risk factors and cancer development

• What are the barriers to cancer risk behaviour change and how can positive behaviour change be encouraged?

• What is the need in Australia for a rare cancer registry to increase volume of and ease of access to information about these cancers?

• What are the perceptions of cancer in culturally and linguistically diverse (CALD) communities?

• How can barriers to prevention be removed and cancer awareness raised amongst CALD groups?

• How can young people be better supported to understand cancer risk?

• Further research into cancer clusters

• Further research into environmental risk factors for cancer development

• What are the most effective interventions for dispelling myths and misconceptions about the causes of cancer in our community?

• Impact of technology on cancer risk – eg mobile phones, CT scans, etc.

• Degree of impact of multi risks in causing cancers

• Do prevention messages cause unnecessary feelings of guilt and self blame?

What is best use of public health dollars re cancer prevention?

Screening and diagnosis

• How can behaviour be changed to improve cancer screening rates?

• What steps are needed to increase skin cancer screening?

• What are the most effective channels for consumers to access information about cancer?

• What is the cancer screening benefit for 70^+^ age group

• What is the scale of exposure to diethylstilbestrol in the Australian community

What are the perceptions and barriers to cancer screening among different cultural groups?

• What interventions work best to increase cancer screening in different cultural groups?

• What interventions work best to increase cancer screening in indigenous groups?

• What are the best approaches to meet the cancer information needs of different groups?

• Investigate genetic testing services in Australia to determine and compare service effectiveness across different population groups and localities

• What is the role and capacity of the general practitioner in encouraging patients to undertake cancer screening?

Is general practitioner training to detect early signs and symptoms of cancer adequate?

• What constitutes a supportive workplace when cancer is diagnosed?

• How can metastatic cancers be diagnosed earlier, preferably before symptomatic?

Treatment

• More investigation into targeted therapies and personalised medicine for all cancers

• Which cancers will benefit from development of immunotherapy?

• Identify best practice treatment protocols and guidelines for all rare cancers

• Overview of research relating to patient treatment profiling for all cancers

• Which drugs available overseas but not to Australians should be fast tracked?

Determine which advanced cancers have yet to have treatment guidelines and address

• Are elderly Australians offered adequate cancer treatment & management?

• What is the difference in cancer treatment decisions of those living in rural, regional and remote areas?

• What are the preferred treatment choices of CALD and Indigenous groups in Australia compared with best practice management?

• Is access to best practice cancer treatment determined by cost in Australia? What impact does this have on health outcomes?

• Investigate the extent and causes for the under representation of particular groups in cancer clinical trials i.e. older people, rural, minorities etc.

• What are the levels of access to cancer clinical trials for residents of rural Australia?

• Is there equity in cancer treatment throughout Australia?

• How can people living in rural areas be best supported emotionally and financially to receive best practice cancer management?

• How can elderly cancer patients be best supported in self management?

• What are the best approaches that health care providers can use to prevent or alleviate patient guilt at cancer diagnosis?

• How can medical care providers be best trained to deliver a cancer diagnosis that is aligned with the patient and family’s level of understanding, emotional and support needs?

• How effective is the communication between different the medical specialists for those with cancer and unrelated co-morbid conditions?

• To what extent are general practitioners ‘kept in multidisciplinary care loop’ of cancer patients?

• What value does multidisciplinary care provide cancer patients?

• What is the currency of cancer specialist and General Practitioner evidenced based knowledge?

• What is the current and predicted need for additional geriatric oncologists in Australia?

• More clinical trials investigating the effectiveness of complementary therapies in cancer control

• How can greater awareness of clinical trials in patients and clinicians be better supported?

• How user friendly and accessible is cancer patient prescription drug information?

• How can we maximise knowledge transfer among researchers working on different cancer types and research disciplines?

• What are the training needs of personal carers of cancer patients and the current availability of such training?

• What are the variations in response to pain management?

• Identify more effective pain management for cancer patients

• Identify individualised pain management interventions

• How can chemotherapy be made less toxic?

• How can the side effects of treatment be better managed?

• What are the different needs of men caring for women with cancer; and women caring for men with cancer?

• What is the extent of carers of cancer patients who are socially isolated and what are their service and support needs?

• What is the extent of carers of cancer patients who are depressed and what are their service and support needs?

• Survivorship

• How can the long term effects of chemotherapy be best addressed?

• What are the particular needs and wants of people living with cancer who do not have adequate social capital?

• What is the level of fear of cancer recurrence among survivors?

• How can the normalisation process be supported (helping those affected by cancer get back to normal)?

• What are the long term effects of repeat diagnostic or prognostic scans for cancer survivors?

• More studies on the effects of chemotherapy on long term cognition

• What is the extent (and intervention availability) of male and female fertility issues post cancer treatment?

• What are the best approaches that can be used to establish and sustain cancer support groups in rural areas?

• Is cancer survivorship related to a greater chance of personal relationship breakdown?

• How can children caring for adults with cancer be best supported?

• What information to survivors need and who should provide it?

End of life care

• What is the extent of use of best practice palliative care guidelines – for patients, carers and family? Gaps?

• What are cancer patients' attitudes to dying with dignity and control?

• Is access to good information about end of life issues (carer, practical, financial, legal, psychosocial), adequate?

• What are the most effective interventions to ensure palliative care service availability in rural and remote areas?

• What is the acceptability and impact of a professional support person in improving the experience and outcomes of those with advanced cancer?

• What extent do different social environments impact on end of life care?

• What are the long term affects and counselling and support needs of carers after the death of a loved one?

• What is it that people value about dying at home? What is it they fear about dying elsewhere?

• What are the needs and areas of unmet needs for respite care for patients and families?

• What is the impact of differing views about end of life, of patient and carer, if any?

### Questionnaire

Fifty seven (19%) of CVN members completed and returned the questionnaires. The majority of respondents were female (56%) aged between 61–70 years (41%). The large majority of respondents reported that the research needs identified at the forum reflected either all or some of their needs (98%) with almost 60% stating they reflected all their research needs. One third of members (33%) identified the research question ‘*how can metastatic cancers be diagnosed earlier before they are symptomatic*?’ as their greatest research need. The second highest priority research questions (32%) were found to be *‘what is the extent of use of best practice palliative care guidelines?’ and ‘more research into nutrition and lifestyle risk factors for people with cancer’* Other high priority research areas include identifying the barriers to cancer risk behaviour change and research into environmental risk factors.

The most common research priorities across all respondents’ first to fifth selections were:

• *‘How can the long term effects of chemotherapy be best addressed?’* (selected in the top five priorities of 77% of respondents)

• *‘How can behaviour be changed to improve cancer screening rates?’* (selected in the top 5 priorities of 75% of respondents) and

• *‘How can the normalisation process be supported (helping those affected get back to normal)?’* (selected in the top 5 priorities of 68% of respondents).

## Discussion

This exploratory project facilitated identification of cancer consumers' priority research needs over five topic areas reflecting major cancer control domains. These were found to be earlier diagnosis of metastatic cancers; the extent of use of best practice palliative care guidelines; identifying barriers to cancer risk behaviour change; and environmental and nutrition and lifestyle risk factors for people with cancer. A process for identifying consumers’ research priorities was developed and applied; this may be useful for further investigation in this under-researched area.

The findings of this project should not be considered in isolation. Consumer research priorities are neither better than nor a replacement for priorities identified by health researchers. They are made available via this project and a range of other published investigations to support researchers to both understand prevailing consumer concerns and needs, and to support and inform decisions on what to research. Ideally consumers and researchers should always work together to identify and detail research topics.

The paucity of Australian research in this area necessitated the use of a broad exploratory approach. The main limitation is the generalisability of findings for a larger population of cancer consumers. However asking consumer organisation representatives to collect the research needs of constituents prior to the workshop is likely to have reduced this problem, as is the comparison of findings of this project with those of a much larger UK study. The similarity between our results and the UK study results suggests that our results are comparable to other populations of cancer patients.

A further limitation is the low response rate (19%) to the postal questionnaire. However, we believe that this low number can be explained in part by the belief among numerous CVN members that they had already provided input on the topic in previous forums. We therefore believe that the views of non-responders are not substantially different from responders.

The findings of this project provide the basis for developing a consumer derived cancer research agenda in Australia, and can be used to help inform the strategic direction of cancer research over the longer term. They also provide direction for cancer care, education and information funding. Australia does not have adequate channels through which community members can express their research priorities or through which they can receive information about the broad scope and success of research being made in the fields with which they are concerned. Lack of recognised processes and resources to collect and analyse relevant information from consumers represents an important obstacle to context and culturally sensitive research needs identification in Australia. At the same time, societal expectations of health and medical research are growing.

The study sought to harvest diverse input from cancer consumers on the issues they need addressed through research. Identifying research needs can be challenging. What processes should be used to determine needs? How can this be done in a systematic, ongoing and efficient manner? What timelines for action should be assigned? This paper adds to the small range of available successful approaches that can be used to capture the research priorities of those directly affected. The *Global Café* method is a user-friendly approach for generating meaningful and cooperative discussion within and between different groups.

It has been argued that consumers do not have adequate understanding to identify useful research priorities [8]. This may have been based on observations of unsupported individual community members rather than networked consumers who are members of advocacy and/or support organisations such as CVN. CVN has been developing informed consumer representatives for over a decade, supported by training in advocacy and research, developed in partnership with CCNSW. The information produced by this project is robust and is validated to some degree by comparing the findings with those of the first UK study to involve cancer patients in a research priorities identification process. Support issues, such as helping those affected by cancer get back to normal, were identified in the UK and current project. Cancer risk factors and early detection were also identified as priorities in both investigations, as were cancer information needs, side effects and cancer recurrence.

Decision makers in publicly funded research systems are under growing pressure to improve priority setting processes and to be more accountable for research funding decisions. There is currently no established structure or mechanism in Australian public research funding schemes that allows consumer research needs to be incorporated. Researchers require training to understand and appreciate what is happening in health and health care overall, and with those adversely affected by too few research answers. Long term partnerships with the end recipients of research provides experiential and contextual learning that are the key to ensuring research is of benefit to and is valued by society. Liaising with intellectual colleagues and attending scientific seminars and conferences alone are unlikely to provide insight into the experiences of those with health problems. Researchers need to be inspired by the individuals and families of those in need of the benefits of focussed research.

## Conclusion

Earlier diagnosis of metastatic cancers; an understanding of the extent of use of best practice palliative care guidelines; identifying barriers to cancer risk behaviour change; and environmental and nutrition and lifestyle risk factors for people with cancer, were found to be the most pressing needs of cancer consumers on the subject of cancer research. The findings of this study offer a workable process for identifying the research needs of health consumers and may be applicable to scenarios other than cancer.

In an era where research is making important progress in our understanding of disease causation and avoidance, and identifying real and potential cures, hopes are pinned on every piece of research to drive more progress. Today’s more informed society is eager and able to encourage a purposeful research culture and direction. Hence, we see the need to build and maintain a critical mass of researchers who are competent in partnering with consumer groups, which in turn can offer capable consumer representatives. This partnership will lead to joint needs identification and priority setting, and to performing quality research of societal relevance.

## Competing interests

Neither author has any competing interests to report.

## Authors’ contribution

Both authors contributed to the design of the project. CS undertook the review of the literature, facilitated the workshop, undertook data analysis and was the main author of the manuscript. Representing Cancer Voices NSW, SC brought this consumer initiative to the CCNSW for possible implementation. SC was a working member of the key reference group, assisted in facilitation of the workshop, organised the postal questionnaire, undertook data analysis and contributed to the preparation and editing of the manuscript. Both authors have read and approved the final manuscript.
